# Use of Cone‐Beam Computed Tomography to Teach Postgraduate Residents Veneer Replacement for Precision and Tissue Preservation

**DOI:** 10.1002/jdd.13963

**Published:** 2025-06-20

**Authors:** Pablo Lenin Benitez Sellan, Edgar Garcia Zea, Eduardo Bresciani

**Affiliations:** ^1^ Department of Restorative Dentistry, School of Dentistry Universidad de Especialidades Espiritu Santo Samborondon Ecuador; ^2^ Department of Prosthodontics, College of Dentistry and Dental Clinics The University of Iowa Iowa City Iowa USA; ^3^ Department of Restorative Dentistry, School of Dentistry São Paulo State University Sao Jose dos Campos Brazil

## 1 Problem

Teaching veneer preparation traditionally relies on visual estimation, which can result in excessive removal of intaglio tissue [1, 2]. This challenge is amplified when preparing old veneers, where preserving the underlying tissue is essential for restoration longevity [3]. Conventional models lack anatomical accuracy, limiting the development of precise techniques for veneer preparation [4]. Additionally, the inability to visualize internal tooth structures can lead to overpreparation, reducing the structural integrity of the tooth [4, 5]. Therefore, an approach that enhances spatial awareness and allows for a minimally invasive preparation is needed, particularly when removing composite resin veneers.

## 2 Solution

**FIGURE 1:**
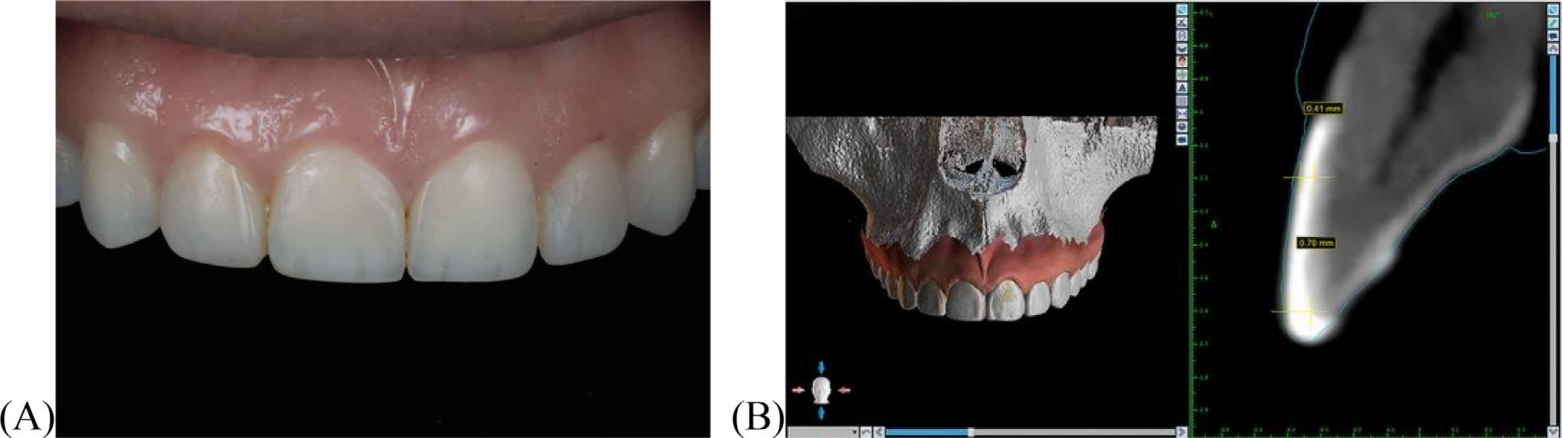
Initial case assessment using cone‐beam computed tomography (CBCT) and intraoral scans. (A) Preoperative clinical condition showing the existing composite veneer; (B) CBCT scan with an external guideline for evaluating the veneer thickness and underlying tooth structure. The blue line represents the external boundary of the dental structure, aiding in precise treatment planning.

To address this issue, cone‐beam computed tomography (CBCT) was integrated into the training workflow. CBCT images of the maxilla were obtained using a CBCT unit (NewTom Giano HR, Italy) from a clinical case involving composite resin veneers, and the images were exported as digital imaging and communications in medicine files. CBCT images and an intraoral scan were imported into design software (BlueSkyBio, USA) to generate 3D reconstructions of the dentition, allowing residents to visualize the veneer, enamel, dentin, and pulp in cross‐sectional views (Figure 1). This visualization enables residents to assess the thickness of each layer and plan veneer preparation while preserving the underlying tissues.

**FIGURE 2:**
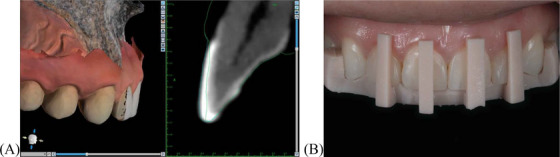
Digital simulation of veneer removal and printed verification guide. (A) Modification of the stereolithography (STL) file to progressively remove the composite resin veneer, represented by the green line, ensuring precise control over the preparation; (B) Printed verification guide designed to monitor the extent of material removal and ensure minimal tooth structure loss during the clinical procedure.

The stereolithography (STL) file of the patient's dentition was modified in the software to simulate veneer removal (Figure 2A). The preparation was adjusted incrementally until the resin layer was digitally eliminated, ensuring minimal impact on the enamel and dentin structures. This process helped residents measure the exact amount of material to be removed, improving precision and conserving the tooth structure. Additionally, the digital slicing feature allowed virtual practice before performing the procedure, followed by the creation of printed verification guides to confirm the amount of tooth structure reduction performed during preparation, thereby enhancing spatial understanding and technique (Figure 2B). These guides were positioned intraorally during the preparation to visually verify alignment with the pre‐planned reduction. A millimeter‐marked periodontal probe was also used to confirm the extent of tissue reduction and its correspondence with the digital design.

Seven postgraduate residents participated in this clinical simulation exercise. Working in pairs under faculty supervision, they collaboratively performed the steps involved in the replacement of six composite veneers across two patients. In some cases, two residents were involved in a single restoration as part of the training protocol. The CBCT scans were originally obtained for clinical diagnostic purposes and were repurposed for educational use, resulting in no additional cost to the patients. Although the software simulation does not replicate the tactile feedback of an intraoral procedure, it provides valuable cognitive and spatial guidance by allowing the user to assess the thickness, angulation, and the limit of preparation virtually.

## 3 Results

**FIGURE 3:**
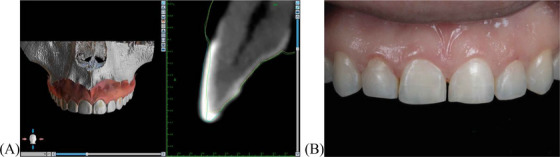
Validation of preparation accuracy using digital analysis. (A) Digital overlay comparison in the software, demonstrating the accuracy of the cone‐beam computed tomography (CBCT)‐guided preparation compared to the initial stereolithography (STL) mesh. The initial state is shown in blue, the simulated preparation on the STL model in green, and the final performed preparation in yellow; (B) Final clinical outcome, confirming that the preparation closely followed the preplanned design, ensuring optimal tissue preservation.

This technique enhances precision by providing detailed anatomical visualization, allowing for a more conservative and predictable approach to the replacement of veneers. A clinical case was performed using a CBCT‐guided preparation plan, applying a digitally simulated removal process. The preparation was performed according to the planned guidelines, and the final result was compared with the initial STL mesh using digital analysis (Figure 3). The comparison showed a high level of accuracy in replicating digital preparation, confirming the effectiveness of the CBCT‐guided approach. The printed guides can also be designed to include proximal contours, which may assist in guiding preparation in interproximal areas.

Residents reported increased confidence in their preparations and a better understanding of tooth morphology and preparation limits. Faculty observations were anecdotal and based on clinical supervision during the case. These improvements were particularly noticeable as the residents involved were at an intermediate training level, and the structured use of digital planning demonstrated progressive refinement of their preparations. Future studies comparing this method with conventional techniques are encouraged to confirm these findings. A limitation of the proposed method is that CBCT imaging may be less effective when evaluating existing ceramic restorations. Materials such as lithium disilicate or zirconia can cause image scattering, depending on their thickness, which may impair the visibility and precision of the planning process.

## References

[1] M. O. Ahlers , G. Cachovan , H. A. Jakstat , D. Edelhoff , J. C. Roehl , and U. Platzer , “Freehand vs. depth‐gauge Rotary Instruments for Veneer Preparation: A Controlled Randomized Simulator Study,” Journal of Prosthodontic Research 68, no. 3 (2024): 407–414, 10.2186/JPR.JPR_D_22_00317. 37853627

[2] Q. J. Wu , X. Wang , and D. Jin , “Validation of Digital Evaluation in Systematic Training on Tooth Preparation in Aesthetic Veneer Rehabilitation,” Chinese Journal of Dental Research 24, no. 1 (2021): 55–60, 10.3290/J.CJDR.B1105879. 33890456

[3] R. L. S. Sanchez , G. G. Dos Santos , G. L. Fukuoka , and I. N. R. Dos Reis , “Replacement of Unsatisfactory Ceramic Veneers With the Aid of a Digital Workflow,” BMJ Case Report 17, no. 5 (2024): e259491, 10.1136/BCR-2023-259491. 38749520

[4] A. J. Cresswell‐Boyes , G. R. Davis , A. H. Barber , M. Krishnamoorthy , and S. R. Nehete , “An Evaluation by Dental Clinicians of Cutting Characteristics and Haptic Perceptions in 3D‐printed Typodont Teeth: A Pilot Study,” Journal of Dental Education 89, no. 4 (2025): 566–577, 10.1002/JDD.13749. PMC12004343 39444145

[5] U. Alexey , S. Franziska , B. Florian , and H. Jeremias , “3D‐printed Educational Model for Anatomy‐driven Preparation and Retraction Cord Placement,” Journal of Dental Education ahead of print, January 6, 2025, 10.1002/JDD.13819. PMC12728785 39763296

